# Mitochondrial Complex I, a Possible Sensible Site of cAMP Pathway in Aging

**DOI:** 10.3390/antiox12020221

**Published:** 2023-01-18

**Authors:** Anna Signorile, Domenico De Rasmo

**Affiliations:** 1 Department of Translational Biomedicine and Neuroscience, University of Bari Aldo Moro, 70124 Bari, Italy; 2 Institute of Biomembranes, Bioenergetics and Molecular Biotechnology (IBIOM), National Research Council (CNR), 70126 Bari, Italy

**Keywords:** cAMP, complex I, signaling, mitochondria, aging

## Abstract

In mammals during aging, reactive oxygen species (ROS), produced by the mitochondrial respiratory chain, cause oxidative damage of macromolecules leading to respiratory chain dysfunction, which in turn increases ROS mitochondrial production. Many efforts have been made to understand the role of oxidative stress in aging and age-related diseases. The complex I of the mitochondrial respiratory chain is the major source of ROS production and its dysfunctions have been associated with several forms of neurodegeneration, other common human diseases and aging. Complex I-ROS production and complex I content have been proposed as the major determinants for longevity. The cAMP signal has a role in the regulation of complex I activity and the decrease of ROS production. In the last years, an increasing number of studies have attempted to activate cAMP signaling to treat age-related diseases associated with mitochondrial dysfunctions and ROS production. This idea comes from a long-line of studies showing a main role of cAMP signal in the memory consolidation mechanism and in the regulation of mitochondrial functions. Here, we discuss several evidences on the possible connection between complex I and cAMP pathway in the aging process.

## 1. Introduction

During aging, reactive oxygen species (ROS), produced by the mitochondrial respiratory chain, cause the oxidative damage of macromolecules leading to respiratory chain dysfunction, which in turn increases ROS production. Several forms of neurodegeneration, and other common human diseases, are often associated with mitochondrial dysfunctions, oxidative stress and aging [1,2]. Therefore, attempts to modulate ROS levels have been carried out in order to understand the role of oxidative stress in aging and aging related diseases. Mitochondrial free radical production is considered to be one of the main causes involved in the determination of longevity [2]. The complex I of the mitochondrial respiratory chain is the major source of ROS production [3,4] and the mitochondrial ROS production, specifically at complex I, has been proposed as one of the major determinants for longevity and is involved in several age-associated neurological diseases [2,5]. 

The cAMP/PKA pathway has a role in the regulation of the respiratory chain activity [6,7,8,9] and in particular complex I [10,11,12], ROS production [10,13] and mitochondrial biogenesis [14,15]. In addition, it has been found that several anti-aging molecules such as the hydroxytyrosol of olive oil [16] and the resveratrol of red wine [17], modulate cAMP pathway, respiratory chain activity and mitochondrial biogenesis [17,18,19]. The cAMP/PKA system appears also to have a role in several neurodegenerative diseases associated with mitochondrial dysfunction, oxidative stress and aging, such as Parkinson’s disease (PD) [20,21], Alzheimer’s disease [22,23] and Down syndrome [24]. It is noteworthy that the activation of the cAMP pathway restores the mitochondrial activity and lowers the ROS level in the mentioned diseases [20,21,24,25,26]. In addition, all members of the cAMP pathway are present in the mitochondria [27,28,29,30], where they can regulate mitochondrial complex I [31], complex IV [32] and ATP synthase [33] activities, ATP production [34], ROS production [32] and susceptibility towards apoptosis [35]. 

Both complex I and cAMP system are strictly connected to aging processes. Herein, evidence on the possible connection between complex I and cAMP pathway in aging are reported. 

## 2. Mitochondrial Complex I

**Figure 1:**
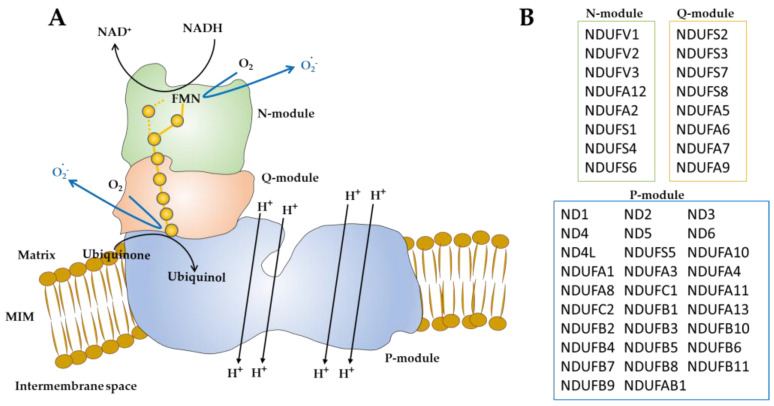
Schematic representation of mammalian complex I. (**A**) Complex I with “L” shape is shown in three distinct modules: N-module (in green), Q-module (in pink) and P-module (in blue). N and Q modules constitute the hydrophilic arm of complex I, exposed in the matrix [45]. The P-module constitutes the hydrophobic arm of the complex embedded in the mitochondrial inner membrane (MIM) [45]. The oxidation of NADH by FMN generates the electrons transfer, through the seven iron–sulfur clusters (yellow spheres), to ubiquinone molecule [44]. The reduction of ubiquinone induces conformational changes in the P-module. This results in proton pumping from matrix to intermembrane space. The reduced FMN, and probably the N2 iron–sulfur cluster, can also react with molecular oxygen (O_2_) to form superoxide anion (O_2_^·-^). (**B**) The complex I subunits belonging to N, Q and P modules are reported [47,48,49,50,51].

The mammalian complex I is the largest mitochondrial respiratory chain enzyme and it is composed of 45 constituent subunits. Seven subunits are encoded by the mitochondrial DNA, 38 by nuclear genes [36]. 14 subunits are conserved from procaryotes to eucaryotes and constitute the catalytic core that maintains the minimal structure for its catalytic activity. The others 31 subunits are called supernumerary. The function of the supernumerary subunits is largely unknown. Some are involved in the assembly and regulation of the complex [8,36,37,38,39]. Complex I is involved in apoptosis [40] and age-related functional decline [41] as well as in a number of metabolic [38], degenerative [42] and proliferative diseases [43]. It is the entry point of NADH reducing equivalents in the mitochondrial respiratory chain catalyzing the electron transport from NADH to ubiquinone. This process is coupled to proton pump from the matrix to intermembrane space, thus conserving the free energy as a transmembrane potential [44]. The transmembrane potential drives ATP formation by complex V. In complex I, several redox centers have been found (FMN, iron-sulfur clusters, and tightly bound ubiquinone), which transfer electrons from the enzyme-bound NADH to the ubiquinone [44] (Figure 1). The complex I structure has a well-known “L” shape with a membrane hydrophobic arm and a matrix-exposed hydrophilic arm. It can be subdivided into three functional modules named N-module (NADH binding and oxidation), Q-module (electron transfer to ubiquinone) and P-module (proton pumping) [45] (Figure 1). Beyond the entry point of reducing equivalents, complex I is also considered to be the major source of reactive oxygen species (ROS) production [3,4] indeed it can also transfer single electrons to oxygen with formation of oxygen superoxide [41,46]. Complex I has been found to produce ROS at FMN and probably iron sulphur cluster N2 [47,48,49,50,51] in the hydrophilic arm (Figure 1).

Several conditions can generate an increase of complex I-dependent ROS production, for example, an excessive reducing power can result in the overproduction of ROS in the mitochondrial matrix with oxidative damage of mitochondrial DNA and subunits of complex I exposed to this space. Complex I can produce anion superoxide during forward electron transfer [4] but it becomes the most important site of superoxide production within mitochondria during reverse electron transfer (RET) [52]. RET is the process, driven by the inner mitochondrial membrane potential, by which electrons from the reduced ubiquinone pool (supplied by succinate dehydrogenase, glycerol-3-phosphate dehydrogenase, electron-transferring flavoprotein or dihydroorotate dehydrogenase) pass through complex I reducing NAD^+^ to NADH [52].

Complex I is a pacemaker of the oxidative phosphorylation system for ATP production [53,54] and its functional capacity is regulated, at transcription and post-translational levels, in response to a variety of regulatory factors/events [8,9,14,38]. In mammals, complex I biogenesis depends on the coordinated expression of mitochondrial and nuclear genes and involves nucleus/cytosol/mitochondria protein traffic and the stepwise assembly of a 45 subunits oligomer [37]. In addition to “de novo” assembly of complex I from all the subunits, an exchange of individual subunits, between the newly imported ones and the older already assembled in the complex, can also occur [37,55]. This process can play a proof-checking role of the subunits in the complex, in particular those superficially exposed to the matrix space, where they can be oxidatively-damaged by ROS, with depression of the catalytic activity. The content of complex I is determined, as for all proteins, by the balance between protein expression and proteolysis. This turnover is particularly critical for complex I because the subunits of its hydrophilic arm are directly subjected to oxidation in the mitochondrial matrix by ROS produced by the complex itself. The functional capacity of complex I appears also to be linked to mitochondrial dynamics [1]. Moreover, the complex I can assemble at higher order with complex II, complex III and complex IV to form supercomplexes [56] in order to modulate the capacity and efficiency [57,58,59] of respiratory chain and the ROS production [59]. 

## 3. Mitochondrial Complex I in Aging

In mammals, a functional decline of mitochondrial respiratory chain activity and thus of complex I has been reported during the aging process, associated with an increased ROS production [41]. For example a study of human skeletal muscle of 63 orthopedic patients of age ranging between 17 and 91 years showed a statistically significant decrease with aging of mitochondrial oxygen consumption with pyruvate plus malate, succinate and ascorbate plus TMPD associated with an age-dependent decrease of the enzymatic activity of complex I, II and IV [60]. It has been suggested that the aging-dependent decline of complex I activity is caused by a decreased expression and increased oxidation of complex I subunits [61,62]. In agreement, several neurodegenerative diseases, such as Parkinson’s disease, are also associated with a decrease of complex I activity and content and an increase of mitochondrial ROS production and complex I subunit oxidation [1,26,42,63,64]. The free radical theory of aging postulates that mitochondrial ROS production causes oxidative damage of macromolecules leading to mitochondrial respiratory chain dysfunction which, in turn, increases ROS production [5]. The complex I, together with complex III, of the mitochondrial respiratory chain is considered the major source of ROS production [41]. Some redox centers, such as FMN radical and ubiquinone in complex I, can be directly oxidized by dioxygen with the single electron transfer to O_2_ and the production of oxygen superoxide [4,46]. Oxygen superoxide can directly oxidize proteins, lipids and nucleic acids. However, complex I produces ROS mainly during reverse electron transport in which electrons pass through complex I reducing NAD^+^ to NADH [52]. This can happen in the condition of reduced ubiquinone, accumulated succinate and higher proton motive force [52]. 

Mitochondrial ROS production, specifically at complex I, is considered to be one of the main causes involved in the aging process and has been proposed as one of the determinants for longevity of species [41,65,66,67]. Indeed, a comparative approach of complex I in the heart tissue of eight different mammalian species with a longevity ranging from 3.5 to 46 years showed a specific difference in the gene and protein expression of complex I related to longevity, and in particular that longevity phenotype is associated with low protein abundance of matrix hydrophilic subunits NDUFV2 and NDUFS4 of complex I [68]. These differences were also associated with reduced ROS levels [68]. Accordingly, a low rate of mitochondrial ROS production has been observed in long-lived organisms, invertebrates and vertebrates, with respect to short lived [66,67]. At the molecular level, the oxidation of mitochondrial DNA and membrane lipids appears to be the traits that correlate with longevity across species. In fact, a negative correlation between mitochondrial DNA oxidation, lipid peroxidation and longevity has been observed, that means a lower oxidation in long- than in short-lived animals even in the same species [69,70].

In the last years, interesting evidences have been provided on the role of content of complex I and ROS production, on the lifespan. The content of complex I, and, in particular, that part of complex I responsible for ROS production, appears to positively correlate with ROS production [71]. Importantly, in mouse and rat, nutritional or pharmacological treatments such as protein or methionine restriction, rapamycin or metformin (inhibitor of complex I) treatment, resulted in higher longevity associated with a decrease of complex I-dependent ROS production [69,72,73,74,75] in heart, skeletal muscle, liver and brain [76]. Moreover, dietary restriction has been also associated with a decrease in the content of complex I [65].

As mentioned above, the longevity-related ROS production by complex I has been ascribed to the peripheral arm [71], and thus to FMN and/or FeS centers [47,48,49,50,51]. In line with this, a study showed that mice under dietary restriction activate an adaptive response leading to a decrease of respiratory chain subunits and, in particular, those that belong to the hydrophilic peripheral arm of complex I [71]. This change has been associated again with a decreased superoxide production at complex I and the improvement of the complex assembly [71]. On the contrary, the inhibition of complex I assembly increased mitochondrial ROS production accelerating aging [71]. The data of the abundance of complex I hydrophilic peripheral subunits have been associated with ROS level [71]. In particular, it has been shown that young longer living mice had a low level of subunits of the hydrophilic arm of complex I compared to old or shorter-living mice, while the hydrophobic arm subunits levels remained unaltered [71]. The authors propose that if the amount of hydrophilic arm subunits is higher than the amount of the hydrophobic arm subunits, the hydrophilic subunits can also assemble into a subcomplex that is able to oxidize respiratory substrate without coupling the proton pumping. This process leads to ROS production that, in turn, accelerates the aging process [71]. This is in agreement with the metabolic labeling study in mammals showing that hydrophilic arm subunits of mitochondrial complex I have a shorter half-lives than hydrophobic arm subunits in liver and heart [77] and in HEK cell line [78]. This supports the hypothesis that the hydrophilic arm subunits of complex I might exist as free [31] or less stable monomer [71] in according also to the finding showing that the new-imported subunits of the hydrophilic peripheral arm of complex I can be assembled in the complex I in exchange with the “older oxidated” ones already assembled in the complex [55,79]. Considering the increase of oxidative stress and the oxidation of complex I subunits during aging [61,62], the continuous import and exchange of the new-synthesized subunits of peripheral hydrophilic arm with the old oxidized ones could represent a mechanism of scavenger of complex I. The levels of hydrophilic arm subunits of complex I appear to correlate positively with the chaperon function of prohibitin proteins (PHB1 and PHB2). Interestingly, in *C. elegans*, the depletion of PHB increases lifespan in mutants with compromised respiratory chain [80] and, in cell with a damaged assembly of complex I, the PHB knockdown restores the assembly lowering the complex I-dependent ROS production [80]. All data are consistent with the possible dangerous effect due to the abundant hydrophilic arm subunits of complex I that can assemble to form a sub-complex producing superoxide, without contributing to proton pumping but contributing to age-dependent mitochondrial decline [71]. In this scenario, it would be very interesting to understand if the specific complex I subunits of the hydrophilic arm are mainly involved. In higher eukaryotes, both NDUFV2 and NDUFS4, a core and a supernumerary subunits, respectively, have been found to negatively correlate with increased longevity [68], instead, the down-regulation of NDUFS2, a core subunit, did not appear to affect longevity [81]. 

In this regard, evidences have been produced in *C. elegans*, in which the knock-down of complex I subunits affected the lifespan [82,83]. In particular the RNA interference of nuo-2 [82,84], the *C. elegans* homolog of mammalian NADH dehydrogenase iron-sulfur protein 3 (NDUFS3) and D2030.4, the homolog of mammalian NADH dehydrogenase 1 beta subcomplex subunit 7 (NDUFB7) [83] extended lifespan leading to a 50% reduction of the ATP production [82,83]. However, the relationship between electron transport chain function, ROS production and longevity appears to be not so simple. Indeed, the timing of RNAi knockdown is fundamental. For example, the reduced subunit expression during adult stages, after completion of the critical L3/L4 larval stages, caused a reduced ATP level that is not, however, associated with an increase of longevity [82]. Moreover, other studies in *C. elegans* have produced evidence showing that worms with reduced electron transport chain activity, induced by the RNAi knockdown of Nuo-1, orthologous to human NDUFV1, of nuo-6, orthologous to human NDUFB4, have extended longevities associated, this time, with an increase of ROS levels [85,86,87]. 

Further studies have been carried out in Drosophila. In line with those observed in *C. elegans*, the reduced expression of two different complex I subunits in flies such as CG9172, the mammalian NADH dehydrogenase iron-sulfur protein 7 (NDUFS7) and CG9762, the mammalian NADH dehydrogenase 1 beta subcomplex 5 (NDUFB5) caused increased lifespan [88]. However, the knocking down of other complex I subunits in flies showed conflicting results in terms of ATP level [88,89] and longevity [89] not reproducing the results observed in *C. elegans*. 

Thus, the attempt to correlate the content of matrix subunits of complex I and the ROS production with longevity is more complicated than expected. Very interestingly, recent findings showed that increasing ROS production, specifically through RET activity at complex I, delays aging and the onset of age-related diseases in flies [90]. In line with this, the suppression of ROS production via RET reduces survival [90]. It has been found that the ROS produced via RET required an uninterrupted electron flow through the electron transport chain that was lost in aged flies [91]. It is important to keep in mind that complex I can assemble together with the complex II, complex III and complex IV to form a supramolecular structure called supercomplex. Of note, the supercomplex formation has been associated with the reduction of complex I-dependent ROS production also via RET [59,92] and a decrease of supercomplex assembly during aging has been observed in mitochondria isolated from the cortex of young (5 months old) and aged (30 months old) Wistar rats [93]. In this scenario, further studies should take in account the ROS production during both forward and reverse electron transfer and the assembly of complex I into supercomplex in order to shed new light on the molecular mechanism of mitochondrial ROS production during aging. 

## 4. cAMP-Dependent Regulation of Mitochondria and ROS Production

cAMP is generated from ATP by adenylyl cyclases (ACs) and degraded by phosphodiesterases. In mammals, there is one gene encoding for multiple isoforms of soluble adenylyl cyclase (sAC) and nine genes encoding for transmembrane adenylyl cyclases (tmAC). tmACs are localized to the plasma membrane, while sAC is present in the mitochondrial matrix and in other organelles [27]. cAMP-dependent protein kinase A (PKA) is a cAMP downstream effector. It is a tetramer that consists of two cAMP-binding regulatory (R) subunits and two catalytic (C) subunits, which dissociate once cAMP binds R-PKA. R-PKA are associated to sub-cellular membranes by binding to specific A-kinase anchor proteins (AKAP). AKAP protein can also bind phosphatases and also phosphodiesterases [94]. In this way, these complexes accomplish activation/deactivation of the signal and phosphorylation/dephosphorylation of substrate bound or close to the complexes modulating the activity of specific proteins [94]. All these proteins involved in reversible cAMP-dependent protein phosphorylation are also present in mitochondria [27,28,29,30]. 

First evidence on the cytosolic cAMP-dependent regulation of complex I comes from Papa’s laboratory showing that the activation of cAMP pathway by widespread addition of a permeant derivative of cAMP (dibutyryl-cAMP or 8-br-cAMP) or by the specific activation of tmAC by β-adrenoceptor agonist isoproterenol or by cholera toxin, results, in a variety of murine and human cell cultures, in the elevation of the NADH-ubiquinone oxidoreductase activity of mitochondrial complex I [8,10,55,95]. This was also associated with a strong reduction of the of ROS level [10,12,13,55,96]. At the same time, the activation of cAMP signaling did not affect the ROS scavenger systems [13,96]. Thus, the cAMP-dependent decrease of ROS level appears to be associated with changes in the enzymatic activity of complex I. The cAMP-dependent promotion of the complex I activity and decrease ROS production are associated to the serine phosphorylation in the conserved RVS site in the carboxy terminus of the subunit of complex I encoded by the nuclear NDUFS4 gene [95,97]. Further insights have been produced on the molecular mechanism of the cAMP-dependent increase of complex I activity. We showed that PKA-mediated NDUFS4 phosphorylation increases its mitochondrial import [98]. Once in mitochondria, the new-imported NDUFS4 assembles in the complex I by replacing the pre-existing, already assembled, oxidized NDUFS4 subunit restoring the activity of oxidatively damaged complex I [55]. Recently, using ex vivo and in vitro models, it has also been shown that the activation of cAMP/PKA cascade resulted in an increase of supercomplex formation associated with an enhanced capacity of electron flux and ATP production rate [58]. This is also associated with the phosphorylation of the NDUFS4 subunit of complex I [58], and at the same time, the formation of supercomplexes has been associated with the decrease of complex I-dependent ROS production [59,92]. 

The cAMP signal also impacts the mitochondrial dynamics by different G proteins. Among these, the Drp1 protein is inactivated by its PKA-dependent phosphorylation. The inactivation of Drp1 results in the inhibition of mitochondrial fission and thus in the promotion of fusion [99,100]. At the same time, the activity of the complex I, as well as of the all respiratory chain, is affected by the balance between mitochondrial fusion and fission, indeed, the inhibition of complex I results in mitochondrial fission [99].

Regarding the mitochondrial pool of the cAMP it has, so far, been found that sAC-dependent cAMP production in mitochondria is involved in the regulation of cytochrome *c* oxidase activity, by direct PKA-dependent phosphorylation of complex IV subunits [32], complex I activity, by regulating the degradation of new-imported subunits of peripheral arm [31] including NDUFV2 and NDUFS4 [31], FoF1 ATP synthase activity and its structural organization [33] and ATP production [34], and in the activation of the mitochondrial pathway of apoptosis [35].

In addition to the post-translational processes the cAMP pathway impacts mitochondrial biogenesis responding to neurotransmitters, cell growth factors, hormones and events like exercise and cold adaptation [101,102,103,104]. cAMP-response element binding protein (CREB) plays a role in the mitochondrial biogenesis [14,105] by promoting the expression of PGC-1α (the transcriptional coactivator PPARγ coactivator 1α [10,95]. PGC-1α, in turn activates the expression of the nuclear respiratory transcription factors (NRF1 and NRF2). NRF1 and NRF2 promote the expression of nuclear genes encoding for proteins of the mitochondrial respiratory chain, FoF1-ATP synthase, mitochondrial import system and enzymes of heme biosynthesis [106]. In addition to the nucleus, CREB is also present in the inner mitochondrial compartment of rat brain where induces the mitochondrially encoded protein synthesis and leads to the increase of complex I-dependent respiration [107,108,109]. 

## 5. cAMP Pathway in Aging

The major complication of normal healthy aging is the increasing risk of age-related diseases [110]. cAMP signaling has been found to be involved in several age-related diseases, such as dementia and metabolic diseases [9,111]. For example, cAMP can impact both insulin and glucagon secretion [112]. Therefore, it is not a surprise that the cAMP pathway also affects diabetes conditions [9]. In this contest, it has been proposed that defects of cAMP pathway contribute to the impaired islet cell function in type 2 diabetes [112]. 

However, although cAMP signaling has been widely studied, little information is available during the aging process. Findings show an altered cAMP signaling associated with physiological brain aging but with conflicting results. Tendentially, in animal brain, a reduction of adenylyl cyclase activity has been found during aging, even if several studies did not reported changes [113,114,115,116]. As for cAMP production, the data on basal cAMP levels are also conflicting depending on mammalian tissues: in white blood cells and serum from human and rodents, cAMP levels were lower in aged than young adult [117,118,119,120]. The basal level of cAMP decreases also in aged rodent cortex [113,115,121,121], thalamus and hypothalamus [115,122], while no changes has been observed in human cerebral microvessels [123,124] and rodent hippocampus [113,115,122,125] and cerebellum [113,115]. Additionally, the PKA activity has been found decreased in the aged prefrontal cortex, hippocampus and serum of rodents and fly brain with respect to young [120,126,126,127]. However, in aged cerebral microvessels, the PKA activity did not change [128]. As for production, basal level and effector, also the degradation of cAMP in aging are widely variable, depending on the specific isoform of phosphodiesterases (PDE) and tissue but, on the contrary of adenylyl cyclase activity and basal level of cAMP, the total PDE activity tendentially augmented with aging [120,129,130,131,132,133,134]. Moreover, it has been shown that the cAMP/PKA signaling is differently affected by aging in the hippocampus and frontal cortex of rats and rhesus monkeys [126,135]. In particular, in newborn rat hippocampus, a low PKA activity has been observed which increases in mature and postmature rats and finally declines in old rats. The most studied protein involved in the cAMP signal during aging process is the protein CREB. CREB protein is ubiquitously expressed in all tissues including brain and it is involved in several functions such as cellular growth, memory and neuronal proliferation. CREB expressed in prefrontal and hippocampus region shows alterations during aging [136]. 

Thus, as general results, even if depending on tissues and species, during aging a decline of cAMP/PKA system appears to occur. 

## 6. cAMP/PKA System and Mitochondria in Aging and Neurodegenerative Diseases

In the last years, an increasing number of studies have attempted to activate cAMP signaling to treat the cognitive decline in age-related diseases associated with mitochondrial dysfunctions and ROS production. This idea comes from a long-line of studies showing a main role of cAMP signal in the memory consolidation mechanism and in the regulation of mitochondrial functions. 

cAMP treatment has been reported to extend the lifespan of wild type Drosophila melanogaster in mice [137]. In particular, in mice it has been shown that exogenous cAMP improved ageing-related phenotypes by increasing sirtuin 3 (Sirt3) protein levels and reducing oxidative stress [137]. Sirt3 is the major mitochondrial sirtuin that regulates complex I activity and ROS production [138]. cAMP, miming the caloric restriction, can increase the protein level of Sirt3 by binding it and preventing its degradation [137,139].

An example of how a deregulation of the cAMP signal results in detrimental impairment of complex I activity associated with an increase of ROS production has been provided by a study in skin fibroblasts of a Down syndrome patient [24]. Down syndrome, caused by trisomy of chromosome 21, is characterized by mental retardation associated with premature aging and neurodegeneration. A strong decrease in the basal cAMP level and PKA activity was found in the patient’s fibroblasts [140] and this was accompanied by a reduction of phosphorylation of the NDUFS4 subunit of complex I, reduced complex I activity and augmented ROS levels [24]. The addition to the cell cultures of dibutyryl-cAMP or epigallocatechin gallate-dependent-activation of the cAMP/PKA pathway rescued all the altered parameters [24]. 

In sporadic PD a decreased activity of complex I and oxidative damage of its subunits have been found in substantia nigra and dopaminergic neurons of autoptic patients [141]. The involvement of complex I is highlighted by exposure to inhibitors of complex I like in drug abusers, in which the MPP^+^ metabolite of MPTP, which inhibits complex I, causes PD [142] and like the exposure of animal laboratories to rotenone that reproduces the characteristic sporadic PD features [143,144]. In NDUFS4 KO mice, resulting in the loss of complex I activity, the dopaminergic neurons are more sensitive to rotenone-induced cell death [143] indicating that defective complex I can be a risk for PD development [143,144]. The mitochondrial dysfunctions have been also observed in familial PD. Mutations of *PARK2* and *PARK6* genes, encoding for parkin and PINK1, respectively, are responsible of the onset of familial PD. Parkin and PINK1 proteins are involved in the mitochondrial quality control pathways in the cells that identifies impaired mitochondria and selectively primes their elimination by mitophagy [145,146]. As for sporadic PD, also in familial PD, a defect of the respiratory chain complex I, decreased ATP production, increased ROS and oxidative stress, and abnormal mitochondrial dynamics have been observed [147,148,149,150]. It has been found, in primary skin fibroblasts harboring mutation in the *parkin* gene, that the mitochondrial dysfunctions were associated with altered cAMP level [21]. In addition, neuroblastoma cell lines, with reduced expression of PINK1, displayed mitochondrial fragmentation and reduced respiratory chain capacity [20,63]. These dysfunctions were rescued by cAMP/PKA system activation [20]. 

In post-mortem samples of brain hippocampal tissues from Alzheimer’s patients, the defective expression of phospho-CREB, PGC-1α, NRF1, NRF2 and mitochondrial transcriptor factor (TFAM) was observed. The same defective expression was observed in APPswe M17 cell line model of Alzheimer diseases and this was rescued by cAMP signal activation in a dose-dependent manner [151]. According to these results, in β-amyloid-treated astrocytes, the activation of cAMP/PKA pathway by glucagon like peptide-1 increased the mitochondrial energy production, the mitochondrial membrane potential and decreased mitochondrial ROS production [26]. 

Very interestingly, in several neurodegenerative diseases such as Alzheimer’s disease, Parkinson’s disease, amyotrophic lateral sclerosis and progressive forms of multiple sclerosis, sharing some common physio pathological mechanisms including mitochondrial dysfunction and oxidative stress [42,64,152,153,154], the use of phosphodiesterase inhibitors that increase cAMP level is strongly under investigation [22,155,156,157]. 

## 7. Conclusions

**Figure 2:**
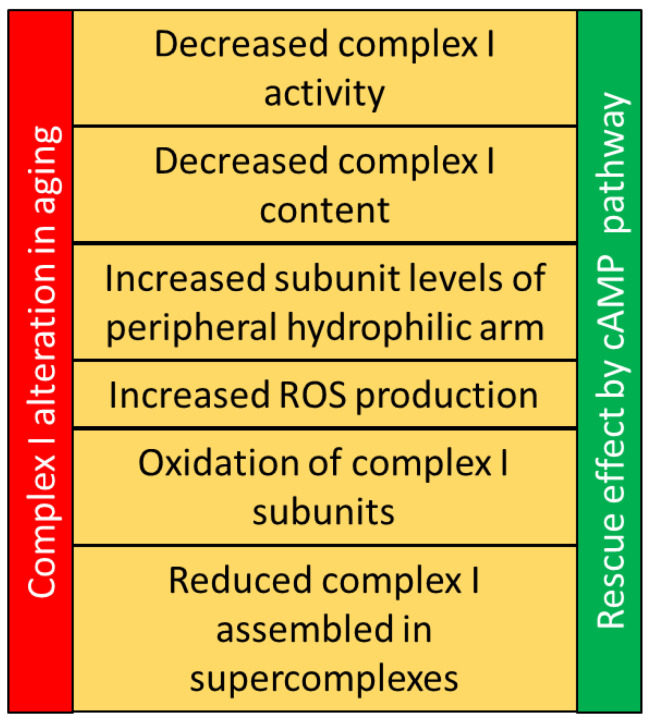
Several alterations have been found to affect complex I during aging process. A functional decline, associated with a decrease of complex I content, has been observed [60,65]. The complex I-dependent ROS production increases with aging in accordance with the increase of oxidation of complex I subunits [2,5,61,62]. Additionally, the superassembly of complex I has been found decreased during aging [93]. Very interesting, a dangerous effect of the subunits belonging to peripheral hydrophilic arm has been observed. In particular, the peripheral subunits hydrophilic arm can form a subcomplex I that oxidize NADH without the proton pumping but producing ROS [71]. cAMP pathway has been found altered during aging process. The cAMP/PKA cascade promotes complex I activity [8,10,55] by increasing the mitochondrial import of the NDUFS4 subunit [98]. Once in mitochondria, the level of the NDUFS4 protein, as well as, the other peripheral subunits, is sustained by mitochondrial cAMP pool [31]. This can allow a dynamic exchange between the new imported subunits and the already assembled probably oxidized ones, as found for NDUFV2 and NDUFS4 [31,37,55]. The PKA-dependent phosphorylation of the NDUFS4 subunit is found associated with the cAMP-dependent increase of complex I assembly in supercomplex [58].

Generally, the aging process in mammals leads to a reduction in genes encoding for the complex I subunits associated with an increase of ROS production (Figure 2). 

Indeed, in age-associated diseases such as Parkinson’s disease, a reduced expression of complex I subunits has been observed to be linked to a decrease of its activity and an increase of ROS production. However, in lower organisms such as *C. elegans* and Drosophila, the decrease of some complex I subunits increases the lifespan and lowers the ROS level. This apparent contradiction could be of note. In fact, in mice, evidences suggest that the hydrophilic arm subunits of complex I can assemble to form a sub-complex producing superoxide without contributing to proton pumping but contributing to age-dependent mitochondrial decline (Figure 2), that means that the increase of this dangerous subcomplex of complex I can favor the aging process. In addition to the role of hydrophilic peripheral complex I subunits, and thus perhaps of a subcomplex, it is worth considering the findings showing that increasing ROS production, specifically through RET activity at complex I, delays aging and the onset of age-related diseases in flies. Moreover, the supercomplex formation has been associated with the reduction of complex I-dependent ROS production, also via RET and a decrease of supercomplex assembly during aging has been observed (Figure 2). 

Importantly, all these processes are regulated at post-translational level in mammals. The cAMP signal is one of the most studied second messenger that impinges mitochondrial complex I functions and content and reduces the complex I-ROS production (Figure 2). Therefore, further research on the aging process could also be directed to the effect of cAMP on mitochondrial ROS production in forward and reverse mode, the subassembly of peripheral hydrophilic subunits and the superassembly of complex I in supercomplexes.

## Data Availability

Not applicable.
